# A Blueprint for the Synthesis and Characterization of Thiolated Graphene

**DOI:** 10.3390/nano12010045

**Published:** 2021-12-24

**Authors:** Maxim K. Rabchinskii, Victor V. Sysoev, Sergei A. Ryzhkov, Ilya A. Eliseyev, Dina Yu. Stolyarova, Grigorii A. Antonov, Nikolai S. Struchkov, Maria Brzhezinskaya, Demid A. Kirilenko, Sergei I. Pavlov, Mihail E. Palenov, Maxim V. Mishin, Olga E. Kvashenkina, Pavel G. Gabdullin, Alexey S. Varezhnikov, Maksim A. Solomatin, Pavel N. Brunkov

**Affiliations:** 1 Ioffe Institute, Politekhnicheskaya St. 26, 194021 Saint Petersburg, Russia; ryzhkov@mail.ioffe.ru (S.A.R.); Ilya.Eliseyev@mail.ioffe.ru (I.A.E.); antonov@mail.ioffe.ru (G.A.A.); Demid.Kirilenko@mail.ioffe.ru (D.A.K.); Pavlov_sergey@mail.ioffe.ru (S.I.P.); Brunkov@mail.ioffe.ru (P.N.B.); 2 Department of Physics, Yuri Gagarin State Technical University of Saratov, 77 Polytechnicheskaya St., 410054 Saratov, Russia; vsysoev@sstu.ru (V.V.S.); alexspb88@mail.ru (A.S.V.); solomatin1994@gmail.com (M.A.S.); 3 National Research Centre “Kurchatov Institute”, Akademika Kurchatova pl. 1, 123182 Moscow, Russia; stolyarova.d@gmail.com; 4 Center for Probe Microscopy and Nanotechnology, National Research University of Electronic Technology, Bld. 1, Shokin Square, 124498 Moscow, Russia; struchkov.nikolaj@gmail.com; 5 Helmholtz-Zentrum Berlin für Materialien und Energie, Hahn-Meitner-Platz 1, 14109 Berlin, Germany; maria.brzhezinskaya@helmholtz-berlin.de; 6 Institute of Electronics and Telecommunications, Peter the Great St. Petersburg Polytechnic University (SPbPU), Polytechnicheskaya 29, 195251 Saint Petersburg, Russia; m.e.palenov@gmail.com (M.E.P.); max@mail.spbstu.ru (M.V.M.); kvol.spbspu@gmail.com (O.E.K.); gabdullin_pg@spbstu.ru (P.G.G.)

**Keywords:** 2D materials, graphene, functionalization, graphene derivatives, thiols, valence band, Mott conductivity, gas sensor

## Abstract

Graphene derivatization to either engineer its physical and chemical properties or overcome the problem of the facile synthesis of nanographenes is a subject of significant attention in the nanomaterials research community. In this paper, we propose a facile and scalable method for the synthesis of thiolated graphene via a two-step liquid-phase treatment of graphene oxide (GO). Employing the core-level methods, the introduction of up to 5.1 at.% of thiols is indicated with the simultaneous rise of the C/O ratio to 16.8. The crumpling of the graphene layer upon thiolation without its perforation is pointed out by microscopic and Raman studies. The conductance of thiolated graphene is revealed to be driven by the Mott hopping mechanism with the sheet resistance values of 2.15 kΩ/sq and dependable on the environment. The preliminary results on the chemiresistive effect of these films upon exposure to ethanol vapors in the mix with dry and humid air are shown. Finally, the work function value and valence band structure of thiolated graphene are analyzed. Taken together, the developed method and findings of the morphology and physics of the thiolated graphene guide the further application of this derivative in energy storage, sensing devices, and smart materials.

## 1. Introduction

The intense studies on graphene oxide (GO) conversion into pristine graphene layers have revealed the inevitable introduction of defects and unintended functionalization accompanying such a process, which often diminishes all the applied efforts [1,2]. In addition to focusing on other approaches for the graphene synthesis, this also triggered the birth of the concept of making the deviations in the chemistry and structure of graphene intended and exploitable to guide its properties in a desired way. Being a giant polyaromatic molecule with a number of different oxygen-containing groups able to undergo substitutional reactions, GO perfectly fits such a goal, converting from a junk graphene counterpart to a versatile platform for the synthesis of graphene-based structures. As a result, the family of chemically modified graphenes (CMGs) covalently functionalized by various groups containing chalcogens or pnictogens has grown dramatically during recent years [2,3,4]. Chemical derivatization appeared to be a facile way to engineer the electronic structure, charge transport, optical absorption, and the material’s response to external effects [4,5,6]. Fluorographene and graphane were found to be wide-gap semiconductors with resistance values beyond GΩ and a bandgap of about 3–3.5 eV [7,8], whereas amination was demonstrated to enhance the conductivity of the reduced GO due to the n-doping from the introduced primary amines, along with the local reduction of the work function value from 4.6 to 4.2 eV [9]. Depending on the concentration and density of the carbonyl or carboxyl groups, the alteration of the conductivity within the range of several hundreds of Ω to MΩ with the substantial modification of the valence band were found for the carbonylated and carboxylated graphene derivatives [10,11,12,13].

Additionally, non-covalent modification of graphene layers with polynuclear aromatic rings via π–π interactions or covalent bonding through the so-called “click” chemistry also is widely studied. Green et al. investigated the modification of graphene by pyrene derivatives by simple mixing of their aqueous suspensions with additional sonication. It was demonstrated that pyrene derivatives anchor on the surface of graphene by π–π interactions and further alter substantially the wetting properties of graphene layers due to the presence of polar functional groups that makes possible to prevent graphene aggregations in various solvents [14]. A well-established and high-yielding method for graphene modification is its functionalization with aryldiazonium salts allowing one to covalently attach simple alkyl and aryl functionalities, as well as complex supramolecular structures, such as cyclodextrins, crown-ethers, and various dendrimer structures [3,4,14,15]. Owing to the inductive and mesomeric effects of the attached polynuclear aromatic rings carrying functional groups, the charge carrier concentration, work function value, as well as absorption and fluorescence spectra can be controllably varied via such a derivatization strategy [4,15,16]. Given these results, CMGs are regarded as the materials of choice for application in field emission devices, photonic devices, such as saturable absorbers or optical limiting materials, as well as sensing devices and blocks for smart materials development [17,18,19].

Graphene functionalization by sulfur-containing functional groups, such as thiols or sulfates, has attracted great attention as well. The introduction of thiol groups has been found to control the nucleation and kinetic stabilization of Pt nanoparticles on graphene as well as carbon nanotubes (CNTs) that advances the promising application of these nanocarbon materials as platinum-on-carbon electrodes for fuel cells [20,21]. Owing to the combination of the conductive nature and electrochemical reactivity of thiols, thiolated graphene enhances a heterogeneous electron transfer rate that is promising in metal-free hybrid electrocatalysts for an oxygen reduction [22]. In turn, Kannappan et al. demonstrated thiolated graphene to be an advanced electrode material for the supercapacitors substantially enhancing their charging and discharging performance at high current density [23]. This is attributed to the fact that thiol groups prevent the re-stacking of graphene layers and the collapse of the pores during cycling.

Functionalization by thiols along with sulfate groups was also applied to controllably tune wetting, mechanical, and electrophysical properties of graphene layers and other nanocarbon-based materials for their subsequent application in functional composites. The thiolation of multi-wall CNTs by microwave plasma chemical vapor deposition was found to enhance field emission properties with a high-field enhancement factor of 1.93 × 10^4^, making such a derived CNTs a promising material for vacuum microelectronics [24]. Regarding fullerenes, thiolation has shown both the important impact of thiols on electron affinity, and, thus, the electrochemical properties of fullerenes along with the governing of their further conversion into orifices and thiafullerenes with one sulfur atom incorporated into the carbon skeleton [25,26]. At the same time, the thiolated graphene have been shown as a facile superhydrophobic sponge for oil–water separation [27], an active layer in the multilayer-structure piezoresistive pressure sensors [28], and an interfacial material in high-performance polymer solar cells [29]. Combining the conductive nature of the graphene layer with the electrochemical activity of the thiol groups towards the adsorption of gas molecules or metal ions has allowed one to consider the thiolated graphene as a promising sensing layer for chemiresistive sensors [30,31].

Still, a synthesis of the thiolated graphene combining the utmost concentration of the covalently bonded thiol groups and restored π-conjugated graphene network with negligible content of oxygen groups, granting advantageous electrochemical and electrophysical properties for practical use, remains an intricate question. The developed methods result in either a low content of the introduced thiols or a poor reduction degree of the treated GO. One of the most common strategies is the co-reaction of GO with thiourea and hydrobromic acid followed by a treatment with sodium hydroxide, in which epoxy and hydroxyls on the basal plane firstly converts into bromine moieties and immediately after into thiols [25,27,28]. However, the achieved concentration of the introduced thiols was only around 1.8 at.% and less. On the other hand, the thiol’s concentration of 5.6% was achieved by Ziółkowski et al. applying the same method, according to elemental analysis measurements [30]. Still, the drawback was a low reduction degree of the treated GO with the absence of the π-conjugated graphene network restoration. GO treatment with a phosphorus pentasulfide dissolved in toluene was proposed by Wrobel et al. as an alternative route for graphene thiolation, but a sulfur concentration of less than 1 at.% was achieved [31]. Similarly, the GO reduction and thiolation via its treating with sodium hydrosulfide hydrate was found to give a high reduction degree with the C/O ratio equal to ca. 25–27, but a negligible concentration of the introduced thiols of less than 1.5 at.% [26].

Thus, an efficient and easy scalable method for the synthesis of thiolated graphene is yet to be developed. In this paper, we report a scalable and facile approach for the production of thiolated graphene via the two-step consecutive bromination and thiolation of GO. The division of these processes to separate stages in comparison to the commonly applied one-step approach allows one to both rise the functionalization degree due to a higher concentration of the intermediate bromine moieties and to achieve an efficient restoration of the π-conjugated graphene network upon Fe-catalyzed GO bromination followed by treatment with thiourea. As a result, thiolated graphene (rGO–Th) is synthesized, which combines the presence of up to 5.1 at.% of thiols and a high degree of the π-conjugation with by the rise of the C/O ratio from 2.3 to 16.8. Taking advantage of the abundance of thiols and the negligible relative number of other groups, the effect of thiol functionalization on graphene’s morphology at a nano- and microscale, as well as its electronic structure and electrophysical characteristics is further thoroughly examined and discussed. This question is often disregarded with the main efforts devoted to successfully synthesizing thiolated graphene for a particular application, while the impact of the thiol groups on the physics and chemistry of graphene is still far from being unveiled. This limits further advances in the engineering of the properties of graphene via thiolation. Collectively, the developed method and the emphasized interplay between the graphene thiolation and its physics allows one to make an advance in further employing this graphene derivative in catalysis, energy storage, and sensing applications.

## 2. Materials and Methods

### 2.1. Materials

An aqueous dispersion of GO was purchased from Graphene Technologies (Moscow, Russia, www.graphtechrus.com (accessed on 20 October 2021). Hydrobromic acid (HBr), 48%, sodium hydroxide (NaOH), iron powder (Fe), Bromine (Br_2_), and thiourea were purchased from Merck KGaA (Darmstadt, Germany). All the organic solvents used in this work were purchased from Vecton Ltd. (Saint-Petersburg, Russia). All the chemicals were of analytical purity grade commercially available. The materials were used as received without additional purification.

### 2.2. Synthesis of rGO–Th and rGO

A two-step method was applied to synthesize the rGO–Th from GO.

At the first step, a reductive bromination of GO was carried out as follows. A GO aqueous dispersion, of 100 mL volume at 0.1 wt.%, was poured into a fluoroplastic flask. Then, sodium silicate powder was added while stirring to reach pH = 11 in the resulting mixture. The pH values of the suspensions were evaluated with a Fisher Scientific Accumet Basic AB15 pH meter (Thermo Fisher Scientific, Waltham, MA, USA). The acquired mixture was subsequently heated at *T* = 80 °C for 24 h in the air. Afterward, the resulting mixture was cooled to room temperature and copiously washed with isopropyl alcohol using a glass filter of 40 μm of pore size. The obtained sediment was placed into a fluoroplastic flask, in which 1 g of Fe powder was added followed by the drop-casting addition of 20 mL of Br_2_ to prevent the violent boiling of the resulting mixture. Thus, the obtained reaction mixture was heated at *T* = 80 °C for 4 h with the flask connected to the reflux condenser. Finally, the suspension was cooled down to room temperature and the synthesized brominated graphene (rGO–Br) was copiously washed with deionized water and isopropyl alcohol using a glass filter of 40 μm of pore size.

At the second step, the synthesized rGO–Br was converted into rGO–Th. The rGO–Br aqueous suspension, of 30 mL volume at 0.1 wt.%, was poured into a glass flask to be mixed with 900 mg of thiourea and 4.5 mL of HBr. The acquired reacting mixture was further heated up to *T* = 80 °C for 24 h while stirring. Following the further cooling down to room temperature, the mixture was enriched with 6 mL of NaOH, 2 M of concentration, whilst stirring for 30 min at room temperature. Finally, the suspension was washed numerous times with isopropyl alcohol using a glass filter of 40 μm of pore size to remove any residuals and yield rGO–Th powder.

The rGO films were prepared by the thermal annealing of GO films on a desired substrate at 650 °C in the ultra-high vacuum chamber, of *P* = 10^−9^ Torr, for 4 h.

### 2.3. CMGs’ Characterization

To investigate the chemistry, morphology, and electronic properties of the synthesized rGO–Th in comparison with the initial GO and pristine rGO, the continuous films prepared on Si wafers by the drop-casting of the corresponding suspension, of 50–200 μL volume at 0.01 wt.%, with the subsequent drying overnight at room temperature, *T* = 25 °C, were examined. X-ray photoelectron spectroscopy (XPS) and X-ray absorption spectroscopy (XAS) methods were applied to give a hint about the chemical composition of the studied CMGs. X-ray photoelectron and X-ray absorption spectroscopy spectra were acquired at the Russian–German beamline (RGBL) of electron storage ring BESSY-II at Helmholtz-Zentrum Berlin (HZB, Berlin, Germany) using the ultrahigh vacuum experimental station [32]. Prior to the measurements, all the samples were placed into a chamber evacuated down to a pressure *P* = 10^−9^ Torr for 6 h to remove all adsorbates. For each studied CMG, the X-ray photoelectron and X-ray absorption spectra were collected in three different areas of the sample and averaged for the following processing.

Survey X-ray photoelectron spectra were recorded with the excitation energy of 850 eV and energy step of 1 eV, while for core-level spectra these values were 850 eV and 0.05 eV, respectively. All the spectra were acquired and refined by a series of five scans with a subsequent correction of their position in accordance with the binding energy (*BE*) of Au 4f7/2 line, *BE* = 84.0 eV. In the case of low-conducting GO films, the position of the C 1*s* spectra redshifted owing to the charging effect due to the materials’ low conductivity was aligned in a way of their low-energy maximum with the *BE* = 284.7 eV, which refers to the well-known position of the *sp*^2^-hybridized carbon [33]. The O 1*s* core-level spectrum was shifted to the same value of *BE*.

The atomic concentrations of the elements were calculated from the X-ray photoelectron survey spectra considering the relative sensitivity factors: C = 1, O = 2.93, and S(2*p*) = 1.68, Br(3*d*) = 2.84. CasaXPS software (Version 2.3.16Dev52, Casa Software Ltd., Teignmouth, UK) was used for the deconvolution and subsequent quantification of the acquired core-level C 1*s*, Br 3*d*, and S 2*p* X-ray photoelectron spectra. Spectra were fitted with Shirley’s background and set of Gauss–Lorentz functions, 70% Gauss and 30% Lorentz (GL (30)). In the case of C 1*s* spectra, an asymmetric Lorentzian Finite lineshape (0.45, 0.85, 200, 750) was applied to fit the peak of C=C bonds due to its inherent asymmetry in addition to Gauss–Lorentz peaks [33]. During the deconvolution of the Br 3*d* and S 2*p* core-level spectra, the relative intensity of the peaks within each doublet, 3*d*_3/2_ and 3*d*_5/2_ for Br 3*d* and 2*p*_1/2_ and 2*p*_3/2_ for S 2*p*, was considered to be 1.4. The deconvolution procedure was performed until the best agreement between the experimental spectra and their fitting was achieved by applying a nonlinear least-squares routine. Afterward, the C/O ratios and the relative concentration of the carbon atoms in different states were calculated for all the spectra acquired for each sample and averaged to yield the reported values.

C *K* and S *L* near-edge X-ray absorption fine-structure spectra were collected in the total electron yield mode by changing the incident photon energy and simultaneously recording the sample drain current. C *K*-edge X-ray absorption spectra were collected within the range of *hv* = 280–315 eV with a step of 0.1 eV, while S *L* one was measured within the range of *hv* = 160–190 eV with a step of 0.1 eV. The measurements were performed at the beam incidence angle of 54.7°. The as-recorded spectra were further normalized and smoothed according to the conventional processing routine [34].

Raman spectra were acquired in backscattering geometry using Horiba Jobin Yvon T64000 and Horiba LabRAM HR 800 (Horiba Jobin-Yvon, Lille, France) spectrometers. The line at *λ* = 532 nm (*E* = 2.33 eV) of a Nd:YAG laser (Torus, Laser Quantum, Inc., Edinburgh, UK) was used as the excitation source. The laser beam was focused by Olympus MPLN 100× (*NA* = 0.9) objective into a spot with a diameter of less than 1 μm. In order to avoid damaging of the samples, the laser power on the material was limited to 0.4 mW. During deconvolution of the acquired spectra, 4–6 peaks were considered, which correspond to either the Lorentz (*D*, *G*, *D*′ peaks) or Gauss (*D*3, *D*4, C=O/C=C peaks) function.

The morphology of the synthesized rGO–Th compared to the initial GO was studied by a set of microscopic methods. Scanning electron microscopy (SEM) images were acquired using the JSM-7001F microscope (Jeol, Tokyo, Japan), whereas a Solver Pro atomic force microscope (NT-MDT, Moscow, Russia) was applied for the atomic force microscopy (AFM) studies. Transmission electron microscopy (TEM) images and electron diffraction (ED) patterns were acquired using Jeol JEM-2100F (Jeol, Tokyo, Japan) with a point-to-point resolution of 0.19 nm at an accelerating voltage of 200 kV. Samples for these studies were prepared by deposition of GO and rGO–Th platelets from the aqueous and isopropyl suspensions, of 5·10^−5^ wt.% of concentration, on the surface of TEM Cu grids (400 Mesh).

Ultraviolet-visible spectroscopy (UV-vis) studies were performed with the help of a Shimadzu-2450 spectrometer (Shimadzu, Japan) without an integrating sphere. Optical density spectra of the GO and rGO–Th films were recorded in the range of *λ* = 190–950 nm with a step of 1 nm.

Electrical conductivity measurements of rGO–Th as a function of temperature were carried out employing a two-electrode system. The rGO–Th film was deposited onto the surface of quartz substrates with two comb Au electrodes, 80 nm thick, separated by a 250 μm gap. The electrode comb consisted of 4 pairs of electrode bars. Prior to the temperature-dependent sheet resistance measurements, the I–V curves were recorded at [−2; +2] V range via Keithley 6487 picoammeter/voltage source (Keithley Instruments, US) to check the Ohmic behavior of contacts. For the temperature-dependent sheet resistance measurements within the 10–300 K temperature range, the rGO–Th sample was mounted on the cold finger of Janis closed cycle refrigerator system CCS-450 equipped with cryogenic temperature controller (LakeShore model 335, LakeShore Cryotronics, Westerville, OH, USA). The cryostat chamber was evacuated to the high vacuum via Pfeiffer Turbo Pumping System HiCUBE 80 eco (Pfeiffer, Germany). Current-voltage measurements were performed at each temperature point for both bias directions after a temperature equilibrium was reached.

The work function (WF) of GO, rGO, and rGO–Th was determined using a conventional approach based on measuring secondary electron cut-off spectra. Given these spectra, the values of WF were calculated using the following equation: *e*Φ_m_ = *hν* − (*E*_F_ − *E*_SEC_), where *hν* = 130 eV is the photons’ energy, and *E*_F_ and *E*_SEC_ are the positions of Fermi level and cut-off threshold, respectively, both represented in the kinetic energy scale [35].

The measurements of the valence band spectra were performed along with the XPS studies using synchrotron radiation at the incident photon energies of ℏω = 130 eV and the energy step of 0.05 eV. To provide a convenient comparison and to eliminate a stochastic noise, all the spectra were accurately smoothed and normalized to the equal intensity of the dip between O 2*s* peak and higher spectral features.

The size distributions of the GO and rGO–Th platelets were also examined by means of the laser diffraction (LD) method. GO and rGO–Th aqueous suspensions, of 0.01 wt.% of concentration, were examined using a Mastersizer 2000 (Malvern Panalytical, Malvern, Worcestershire, UK) in accordance with the procedure described earlier by Rabchinskii et al. [36].

Dynamic light scattering measurements were carried out to examine the zeta-potential of the GO and rGO–Th in the aqueous media using a Malvern Zetasizer Nano ZS instrument (Malvern Panalytical, UK) at a temperature of 25 °C. Similar to the LD measurements, GO and rGO–Th aqueous suspensions, of 0.01 wt.% of concentration, were investigated.

The chemiresistive properties of the rGO–Th films were evaluated by being placed over the Si/SiO_2_ substrate equipped with multiple electrodes according to earlier reported protocols [12] via drop-casting deposition of rGO–Th platelets from the isopropyl suspension, of 5 × 10^−5^ wt.% of concentration. The inter-electrode distance to measure the material’s resistance was 50 µm. For primary tests ethanol vapors were chosen as analyte test gas, which was further mixed with background dry and humid air served as a reference that is close to real practice applications in difference to N_2_. The detailed description of the measurement setup is presented in Supplementary Materials, Section S1, Figure S1. The chemiresistive response was calculated as a relative change of resistance in percent:

(1) $$S=\frac{R_{eth}-R_{air}}{R_{air}}\cdot 100\%,$$

where *R_air_* is the film resistance in a background air and *R_eth_* is the film resistance in an analyte-enriched air. The vapors were delivered from the corresponding liquid of analytical grade by bubbling in a flow mode at 400 sccm rate managed by high-precision mass-flow controllers.

## 3. Results

### 3.1. Chemistry of rGO–Th

**Figure 1:**
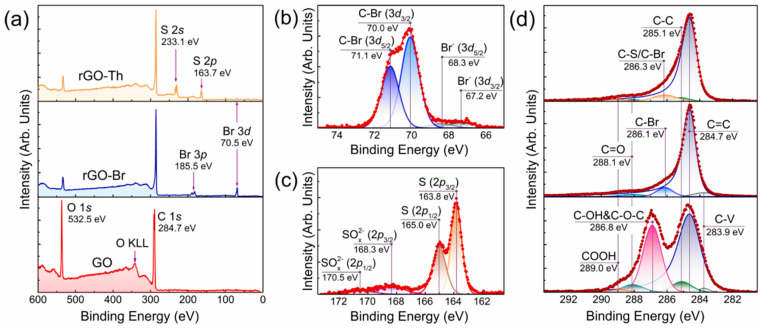
X-ray photoelectron examination of the CMGs’ chemistry. (**a**) Survey and core-level (**b**) Br 3*d*, (**c**) S 2*p*, (**d**) C 1*s* X-ray photoelectron spectra of the initial GO, rGO–Br, and rGO–Th.

Figure 1a displays the survey X-ray photoelectron spectra of the initial GO, rGO–Br, and rGO–Th, giving a hint on the elemental composition of these CMGs. The GO spectrum is comprised solely by O 1*s* and C 1*s* lines with the *BE*s of 532.5 eV and 284.7 eV, respectively, signifying the absence of contaminating species. The dominance of the O 1*s* core-level line implies a high oxidation degree of the initial GO with the estimated C/O ratio to be ca. 1.9. Upon the applied reductive bromination, the O 1*s* signal drastically decreases while both Br 3*p* peak with *BE* of 70.5 eV and Br 3*p* doublet centered at 185.5 eV appear, indicating the successful removal of the oxygen groups and their partial substitution with bromine moieties. The atomic concentration of the bromine species derived from the quantitative analysis of the spectrum was estimated to be ca. 5.9 at.%. To further verify the covalent bonding of the introduced bromine to graphene network, the high-resolution Br 3*d* spectrum was examined after a deconvolution (Figure 1b). Two doublets are discerned: C–Br (3*d*_3/2_) and C–Br (3*d*_5/2_) doublet with *BE*s of 70.0 eV and 71.1 eV, corresponding to bromine atoms covalently bonded to carbon [37,38]; and Br^−^ (3*d*_3/2_) and Br^−^ (3*d*_5/2_) doublet with *BE*s of 67.2 eV and 68.3 eV, arising due to the presence of the physisorbed bromine ions [38,39]. The relative concentrations of the bonded and physisorbed bromine estimated from comparison the relative areas of the corresponding doublets are calculated to be 92% and 8%, respectively. Accordingly, the concentration of C–Br species appears to be about ~5.43 at.%, which is among the highest reported values [37,38,39,40].

Moving from rGO–Br to rGO–Th, an almost complete elimination of the Br 3*d* peak is indicated in the survey spectrum with the simultaneous rise of a pair of peaks with *BE*s of 163.7 eV and 233.1 eV, which come from S 2*p* and S 2*s* core-level lines, respectively [29,41]. The quantitative analysis yields the atomic concentration of S to be at 5.93 at.%, which is beyond the values for the thiolated graphene derivatives published up to date [23,27,28,42]. The presence of the hardly distinguishable Br 3*d* peak is due to the retention of traces, less than ca. 0.13 at.%, of the highly stable C–Br species that remain at the edges of the graphene network. The analysis of the S 2*p* high-resolution spectra after its deconvolution has demonstrated that sulfur moieties are mainly featured by thiols or disulfides, revealing themselves by the presence of the S (2*p*_3/2_) and S (2*p*_1/2_) doublet centered at *BE*s of 163.8 eV and 165.0 eV, respectively [23]. Besides, a minor contribution of the sulfur bonded to oxygen, such as sulfates or sulfites, is manifested by the presence of the discerned doublet of SO_x_^2−^ (2*p*_3/2_) and SO_x_^2−^ (2*p*_1/2_) with *BE*s of 168.3 eV and 170.5 eV, respectively [31]. Given the relation between the areas of the corresponding doublets, the relative content of thiols or disulfides is estimated to be 86% with the corresponding atomic concentration at ca. 5.10 at.%, whereas the rest 14% of sulfur moieties, or 0.83 at.%, are oxidized sulfur species.

The processed C 1*s* X-ray photoelectron spectra displayed in Figure 1d further point out that the bromination and thiolation of the initial GO is accompanied by an intensive elimination of oxygen groups from the basal plane and edges of the graphene layer. Table 1 summarizes the quantitative data on the composition of the studied CMGs derived from the processed C 1*s* spectra. The initial GO is mainly covered by the hydroxyls and epoxides signified by a prominent C-OH&C-O-C peak with the *BE* of 286.8 eV, to be accompanied with the presence of ketones and carboxyls at the edges, which are expressed in C 1*s* spectra by C=O and COOH peaks centered at 288.1 eV and 289.0 eV, respectively [10,12,43]. The relative concentrations of these oxygen species are 37.3 at.%, 4.26 at.%, and 1.26 at.%, correspondingly, yielding the C/O ratio of 2.3. This value is slightly higher than the one estimated from the survey spectrum due to the additional impact of the intercalated water on the O 1*s* signal in the latter case, thus slightly diminishing the C/O ratio.

Upon bromination, the C=O and COOH peaks were lowered while the C-OH&C-O-C peak became unresolvable against a new spectral feature centered at *BE* of 286.1 eV, corresponding to C–Br species [39]. The area of the C–Br peak was restricted in a way to give the calculated concentration of the covalently bonded bromine in the C 1*s* spectrum equal to the one derived from the survey spectrum, implying the self-consistency of the applied deconvolution model. Owing to the reduction of the oxygen groups and a retention of only 2.68 at.% of ketones, the C/O ratio of rGO–Br rose to ca. 37.4. At the same time, the following conversion of rGO–Br into rGO–Th resulted in the lowering of the C/O ratio down to 16.8 with the rise of the COOH peak again. This effect is due to the introduction of the sulfate and sulfite groups along with thiols indicated by the analysis of the S 2*p* core-level spectrum. Inducing almost the same chemical shift as carboxyl groups due to comparable electron-withdrawing effect, the SO_x_^2−^ species provide a rise in the spectral feature at *BE*s of 289–289.4 eV and a slight further O enhancing in rGO–Th compared to rGO–Br. The alterations in the composition of functional groups, covering the basal plane and edges of the graphene layer, are also reflected by the changes in the zeta-potential values of GO and rGO–Th. Upon GO conversion to rGO–Th with the elimination of the negatively charged dissociating oxygen groups, the zeta-potential rises from −60.1 mV, which corresponds to the earlier reported values [44], to −37.1 mV. This value is still lower than the commonly obtained values for pristine rGO, of −15–20 mV [45], and relates to the impact of the introduced thiol groups.

Nevertheless, rGO–Th still possesses a high reduction degree comparable to commonly reported values for the pristine rGOs [43,46]. The recuperation of the π-conjugated graphene system is specified by the dominance and asymmetry of the C=C peak with *BE* of 284.7 eV related to π-bonded carbon atoms. The asymmetry of the peak arises from the screening effect of the π-conjugated system upon the photoionization of electrons from the graphene network and the generation of electron-hole (*e*-*h*) pairs [47]. At the same time, the two other peaks of the non-functionalized carbon atoms, namely the C–V peak centered at 283.9 eV and C–C peak with *BE* of 285.1 eV related to non-conjugated carbon atoms and non-terminated carbon atoms at the edges of vacancies, have a negligible contribution [48].

**Figure 2:**
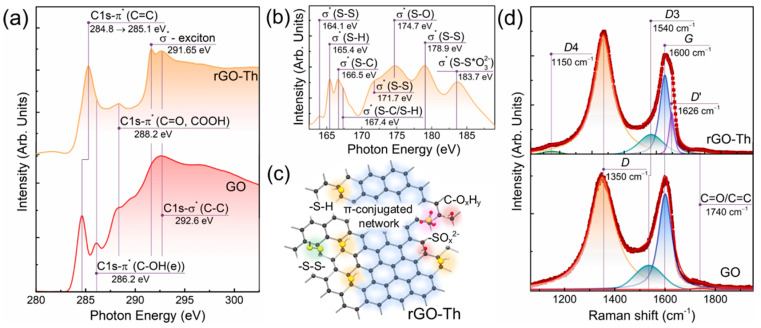
Chemistry and structure of rGO–Th. (**a**) C *K*-edge and (**b**) S *L*-edge X-ray absorption spectra of the initial GO and rGO–Th. (**c**) Schematic representation of rGO–Th. (**d**) Raman spectra of the initial GO and rGO–Th.

The restoration of the graphene electronic structure in rGO–Th is further signified by the comparative analysis of the corresponding C *K*-edge X-ray absorption spectra displayed in Figure 2a. The GO conversion into rGO–Th results in an evolution of the C 1*s*-π* resonance at *hv* = 285. 1 eV, which matures from the electron transitions in the C=C bonds at pristine *sp*^2^-domains [47]. It shifts from *hv* = 284.8 eV to *hv* = 285.1 eV with the increase in its ratio to the σ*-resonance of graphene lattice (C 1*s*-σ* peak) from 0.57 to 0.86. No broadening of the C 1*s*-π* (C=C) resonance is indicated, which is commonly observed in the case of the presence of localized *sp*^2^-domains of varied dimensions having a diverse conjugation length [12,49]. Conversely, the evolution of the C 1*s*-π* resonance is accompanied by the appearance of the σ*-exciton resonance centered at *hv* = 291.65 eV, which is a common sign of the complete recuperation of the extended conjugated domains of the graphene network [50,51]. At the same time, the C 1*s*-π* resonances of the edge-located hydroxyls at *hv* = 286.2 eV [C 1*s*-π* (C-OH(e))] and ketones/carboxyl groups at *hv* = 288.2 eV (C 1*s*-π* (C=O, COOH)) disappear or diminish, verifying the elimination of the oxygen groups indicated by the XPS studies.

S *L*-edge X-ray absorption spectrum was collected as well to delve into the composition of the introduced sulfur species (Figure 2b). Absorption bands related to 2*p*_3/2_ → σ*(S-H) and 2*p*_3/2_ → σ*(S-C) transitions centered at *hv* = 165.4 eV and *hv* = 166.7 eV, respectively, can be discerned [52]. Combined with the presence of an absorption band at *hv* = 167.4 eV comprised by a mix of 2*p*_1/2_ → σ*(S-H) and 2*p*_1/2_ → σ*(S-C) resonances, the S *L*-edge X-ray absorption spectrum justifies the asserted presence of thiols. Furthermore, sulfur species with disulfide bonding reveal themselves by the presence of the σ*- resonances positioned at *hv* = 164.1 eV, *hv* = 171.7 eV, and *hv* = 178.9 eV [53]. The origin of distinguishable absorption bands at *hv* = 174.7 eV and *hv* = 183.7 eV is less unambiguous and attributed to the transitions either to the σ*(S-O) molecular states or empty S 3*d* states [54,55], thus just assuming the aforementioned presence of a certain number of sulfates or sulfites. The observed absence in the dependence of intensity of the absorption bands of thiols, disulfide, and sulfur–oxygen species, is due to a hindered relation between the number of a certain moiety and relative intensity of the corresponding absorption band as a sequence of the strong impact of the orientation and cross-sections the corresponding orbitals as well as the complicated nature of the background [49,50].

Thus, given the XPS and XAS results, rGO–Th is shown to be comprised by the extensive areas of the π-conjugated network locally functionalized predominantly by thiols and disulfide moieties with a negligible content of sulfates/sulfites and retained oxygen groups. Figure 2c highlights the key features of the chemistry of the synthesized rGO–Th.

### 3.2. Morphology and Structure of rGO–Th

To assess the influence of the alterations in the chemistry upon GO conversion into rGO–Th on the morphology and structure of the material, Raman spectroscopy studies were carried out. Figure 2d displays the deconvoluted Raman spectra of the initial GO and rGO–Th films. The spectra are dominated by *D*, *D*3, *G*, *D*′, and C=O/C=C bands centered at *v* = 1350 cm^−1^, *v* = 1540 cm^−1^, *v* = 1600 cm^−1^, *v* = 1626 cm^−1^, and *v* = 1740 cm^−1^, respectively. The *G* band comes from doubly degenerated vibrational *E*_2g_ mode along the C–C bonds, whereas the *D* and *D*′ bands correspond to the full-symmetry vibrational mode of the *sp*^2^-hybridized carbon of graphene network. Both *D* and *D*′ bands indicate the defectiveness of the graphene layer since the corresponding scattering of the phonons with non-zero wave vector requires a defect according to a fundamental selection rule of Raman spectra [56]. The origin of *D*3 band is commonly attributed to the presence of amorphous carbon [57], whereas the nature of C=O/C=C band could either relate to the vibrational mode of benzene rings or C=O bonds in ketones and ethers [58,59].

Upon the GO reduction and functionalization by thiols, the Raman spectrum considerably changes: the full width at half maximum of the *D* band significantly reduces from 120 cm^−1^ to 96 cm^−1^, *D*4 band centered at *v* = 1150 cm^−1^ appears, whereas the *G* band splits with the rise of the *G*′ band positioned at ca. *v* = 1626 cm^−1^. The observed narrowing of the *D* band points out the increase in the extension of the π-conjugated areas [60], supported by rising the *D* band intensity. Particularly, the *D* band evolution is non-monotonic: primarily the *D* band rises, but further starts to decrease due to a higher discordance degree and vice versa for the recuperation of the graphene network [61]. Since the intensity of the *D*′ band is inversely proportional to the content of *sp*^3^-hybridized carbon, its appearance verifies the elimination of the predominant number of oxygen groups, although still indicating the introduction of vacancy defects, graphene edges, or folds [62]. The nature of the *D*4 band is less unambiguous, being attributed to either the presence of polyene chains, *sp*^3^-hybridized carbon, or functionalization by various groups, such as amines [63,64,65]. Considering the absence of this band in the GO spectrum and introduction of thiols, the latter interpretation is regarded as the most relevant one, although further studies are needed on this question.

**Figure 3:**
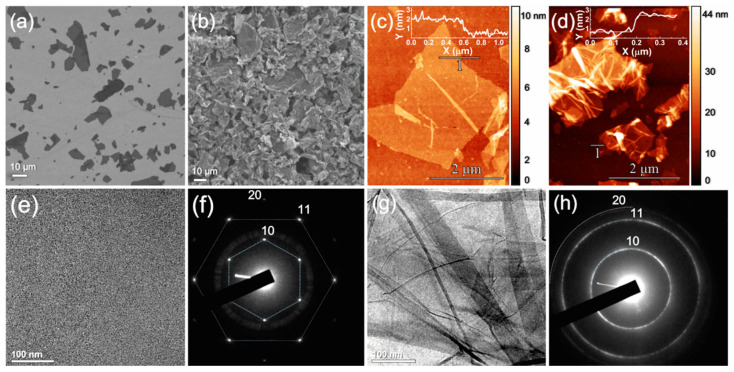
Microscopic studies of the morphology of the initial GO and rGO–Th. (**a**,**b**) SEM and (**c**,**d**) AFM images of the platelets of GO and rGO–Th, respectively. Insert—height profile measured along the line 1. (**e**,**g**) TEM images and (**f**,**h**) ED patterns of GO and rGO–Th, respectively.

Microscopic studies further evidence the difference in morphology between the studied CMGs asserted by Raman spectroscopy. Figure 3 displays exemplary SEM, AFM, and TEM images along with the ED patterns of GO and rGO–Th. Both CMGs are comprised of flakes, exhibiting the irregular shape with the sharped edges and the absence of the distinct lateral anisotropy. The lateral size of both the initial GO and rGO–Th lies within the range of 3–40 μm with the mean equivalent diameter of 6–8 μm, as seen in the SEM and AFM images (Figure 3a–d), combined with size distribution measurements by the LD technique (Supplementary Materials, Figure S2). The thickness of GO and rGO–Th were found to be ca. 1.4 nm and 2 nm, respectively, which confirms the monolayer nature of both graphene derivatives accounting for the excess over the reported thickness of 0.8–1.1 nm due to the presence of modifying groups on the basal plane [66,67]. The slight excess of rGO–Th thickness over GO one can arise from both a distinction in interactions of functional groups with the AFM tip in the tapping mode and a residual solvent layer at the flake/substrate interface. Regarding the morphology of the GO and rGO–Th flakes, the density of the corresponding films is estimated to be 0.74 g/cm^3^ and 1.3 g/cm^3^, respectively. Also, owing to the interlayer disulfide bonding (-S-S-) and π–π interaction between the restored areas of the π-conjugated network without sonication for more than several hours, the rGO–Th platelets tend to stack forming lamellar particles of several layers (Figure 3b).

However, despite the mentioned similarities in platelet structure, the intensive corrugation of the graphene layer upon GO conversion into rGO–Th is indicated. GO platelets, being initially flat and aligned along the surface of the Si wafer (Figure 3a,c), become crumpled after the elimination of the oxygen groups and the introduction of thiols with a high number of disordered folds, which extend throughout the total surface (Figure 3b,d). The average height of such folds is about several tens of nm. The demonstrated crumpling is asserted to mature from the strain induced by the functionalization of the basal plane by isolated sulfur-containing groups. This is supported by comparing SEM images of rGO–Th to the ones of rGO–Br, in which individual bromine species cover graphene layer (Supplementary Materials, Figures S3 and S4). Analogously to rGO–Th, rGO–Br demonstrates a corrugated structure with the curved shape of graphene platelets due to strain induced by non-uniform functionalization by bromine species, inducing graphene lattice distortion. Considering the absence of covalent bonding between bromine species, the curvature of the rGO–Br platelets is even higher compared to rGO–Th being able to interact with the formation of disulfide bonds, which slightly aligns and flattens of the graphene layer. In contrast, in GO, a large number of oxygen groups is uniformly distributed and checkered on both sides of the layer, compensating the induced out-of-plane distortion of each other. Combined with electrostatic repulsion between oxygen groups, this results in the quasi-flat configuration of the graphene layer [68,69].

This assertion is further ratified by comparing TEM images and ED patterns of GO and rGO–Th. Analogously to SEM and AFM, low-magnification TEM imaging of GO displayed in Figure 3e evidences its planar structure with the absence of any rips and folds. The corresponding ED pattern (Figure 3f) is presented by a single set of six sharp (10) and (11) diffraction maxima with detectable (20) diffraction pattern, which collectively indicate a long-range crystalline order with the absence of considerable distortions of the graphene layer as well as verify the monolayer nature of GO platelets [70]. In turn, a large number of folds is shown by the TEM image of rGO–Th displayed in Figure 3g, analogously to folds in the rGO–Br platelets (Supplementary Materials, Figure S5a). The notable wrinkling of rGO–Th as well as rGO–Br at a nanoscale is accompanied by the transformation of the ED pattern into a ring-shaped one due to the rotational shift of the stacked folds and wrinkles of the graphene layers. In the case of rGO–Br, diffraction spots still remain distinguishable (Supplementary Materials, Figure S5b), whereas for rGO–Th, the diffraction spots have almost completely merged into a ring. Along with TEM images, this suggests the larger density of the nanoscale folds in rGO–Th compared to rGO–Br, owing to a higher concentration of thiols. At the same time, no diffusion and blurring of the ring-shaped diffraction patterns, inevitably accompanying the reduction of the long-range order of the graphene lattice at atomic scale [71], is observed together with the retention of a detectable (20) diffraction pattern. Given the absence of the holes or rips indicated in the acquired TEM images, these results imply that, apart from the formation of folds, the graphene network in rGO–Th retains high structural quality with long-range order, coinciding with the results of the XAS analysis.

### 3.3. Optical of rGO–Th

**Figure 4:**
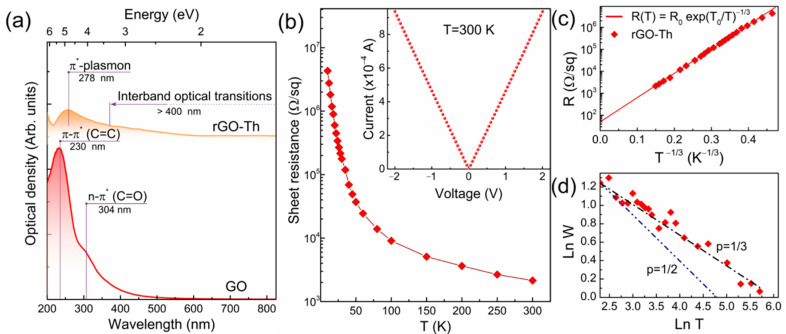
Optical and electrophysical properties of rGO–Th. (**a**) UV-vis spectra of the initial GO and rGO–Th. (**b**) Semi-log scaled plot of resistance versus a temperature. Insert—the I–V curve measured at *T* = 300 K. (**c**) The resistivity *ln R* versus *T*^−1/3^ plot. The symbols are the experimental points, and the solid line is a fit to *T*^−1/3^ approximation. (**d**) Reduced activation energy (W) plotted versus a temperature (T) in a log–log scale; the lines corresponding to *p* = 1/2 and *p* = 1/3 are displayed as a comparative guide for eyes.

Apparently, in addition to the alteration of the graphene layer morphology, the effect of graphene functionalization by thiols on the optical and electrophysical properties along with electronic structure is of high interest regarding the possible application of rGO–Th in the fields of catalysis, energy storage, sensors and optoelectronic devices. Figure 4a displays UV-vis spectra of rGO–Th compared to the one of the initial GO, which is comprised of the main absorption band at *λ* = 230 nm of the π–π* electronic transitions in C=C bonds and a shoulder centered at *λ* = 304 nm attributed to n–π* transitions in C=O bonds with almost complete absence of absorption in the visible range [68,72]. Upon the elimination of oxygen groups and restoration of the extended π-conjugated graphene network, the optical absorption in the visible and near-infrared range of spectra drastically rises, while the 230 nm peak redshifts to ~270 nm, or *E* = 4.46 eV, which is close to the known value of π-plasmon in graphene with the energy of ca. 4.5–4.6 eV [73]. Concurrently, the absorption band that appeared at *λ* = 304 nm is reduced to be commonly interpreted as a result of ketones and carboxyl groups elimination, although the absence of this absorption band has been recently demonstrated to be independent of the concentration of these oxygen groups [12,74]. At the same time, no additional absorption bands that could arise due to the functionalization by sulfur groups are indicated with optical absorption of rGO–Th being equal to the one of pristine rGO.

### 3.4. Electronic Properties of rGO–Th

To further assess the rGO–Th conductivity mechanism and values, two-probe sheet resistance measurement in a wide range of temperatures 10–300 K was carried out. Figure 4b displays a semi-log scale plot of resistance versus temperature measured for the rGO–Th film of 150 nm of thickness. The Ohmic contact between the film and measuring electrodes was verified by the linear behavior of voltage (*V*) on current (*I*) acquired in the range from −2 V to +2 V. The sheet resistance of the rGO–Th layer at room temperature is measured to be ~2.15 kΩ/sq, which is almost of the same magnitude as reported for conventionally synthesized pristine rGOs [75,76]. In turn, upon moving to low temperatures the sheet resistance rapidly grows by almost four orders of magnitude that signifies the semiconducting nature of the rGO–Th film where electron transport is governed by the variable hopping (VRH) mechanism in accordance with the following expression [77]:

(2) $$R\left(T\right)=R_{0}\exp{\left(\frac{T_{0}}{T}\right)}^{p}$$

where *R*_0_ is a pre-factor, *T*_0_ is a characteristic temperature, and *p* is a characteristic exponent whose value distinguishes different types of VRH mechanism [77,78,79]. Depending on structural quality and functionalization parameters, both Mott variable range hopping (Mott-VRH) and Efros–Shklovskii (ES-VRH) conduction mechanisms have been previously distinguished in various CMGs [76,78,79]. Employing a common approach to determine the type of VRH mechanism by plotting *ln R* vs either *T*^−1/3^ (Mott-VRH) or *T*^−1/2^ (ES-VRH), the Mott-VRH mechanism was asserted for the rGO–Th since experimental data almost perfectly fit the *T*^−1/3^ behavior (Figure 4c). However, the difference between *T*^−1/3^ and *T*^−1/2^ is not that substantial. Some recent articles have reported that similar experimental data fitted with both *T*^−1/3^ and *T*^−1/2^ curves, making the accurate analysis of hopping conduction extremely difficult [80]. Therefore, to analyze the type of the VRH mechanism more precisely, the dimensionless energy of activation W has been examined derived as:

(3) $$W=\frac{\partial ln\;R\left(T\right)}{\partial ln\;T}=p\times {\left(\frac{T_{0}}{T}\right)}^{p},$$

The *p* value in this case is obtained from the slope of *ln W* vs. *ln T* plot as drawn in Figure 4d. The presented curve demonstrate that the data follow properly the *p* = 1/3 line with the *p* value estimated from the slope to be ca. 0.31. Accounting for these results, the Mott-VRH mechanism of conductivity is determined for the rGO–Th samples, which complements the spectroscopic and microscopic results on the considerably high structural quality and restoration of the π-conjugated graphene network after the functionalization.

**Figure 5:**
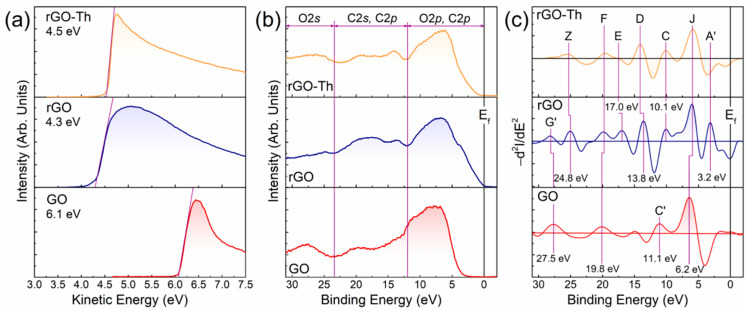
Analysis of the electronic structure of rGO–Th compared to other CMGs. (**a**) Cut-off SE spectra of the initial GO, rGO, and rGO–Th. (**b**) VB spectra and (**c**) their second derivative (−d^2^*I*/d*E*^2^) of the CMGs under study.

The effect of thiol functionalization on the graphene work function and structure of the valence band has been studied as well. Figure 5a displays the SE cut-off spectra of the initial GO, pristine rGO, and rGO–Th. The estimated values for two former CMGs are 6.1 eV and 4.3 eV, which is in good consistency with the literature data [81,82]. In turn, the WF value for rGO–Th is somewhat intermediate between the ones of GO and rGO, being equal to 4.5 eV. This is due to the electron-withdrawing effect of sulfur atoms in thiol groups, which is coupled with the large extension of the recuperated π-conjugated system results in a slightly higher WF of the rGO–Th. Notably, this, in contrast to graphene functionalization by amines, has been demonstrated to reduce the WF of the graphene layers due to the electron-donation effect of aromatic amines [9,83].

### 3.5. Valence Band Structure of rGO–Th

Compared to the WF value, no considerable effect of graphene functionalization by thiols to the VB structure was found. This is in contrast to the recently revealed introduction of new localized states related to the molecular orbitals of ketones and carboxyl groups upon the modification of graphene layer edges by the corresponding functional groups [84]. In the case of rGO–Th, the VB structure almost completely represents the one of pristine rGO (Figure 5b). This is clearly seen from the comparative analysis of their second derivatives taken with the opposite sign and displayed in Figure 5c, which makes possible to unveil broadened, overlapping spectral features and to precisely define their positions [85]. Both the rGO and rGO–Th VB spectra are dominated by a broad band *J* centered at *BE* of ca. 6.2 eV attributed to the 2*p*-σ electron states of graphene network, which is shifted in the case of GO to ca. 6.8 eV due to the localization of π-conjugated domains by the presence of basal-plane hydroxyls and epoxides [86]. Furthermore, a set of maxima of graphene density of states (DOS) denoted as *D*, *E*, and *F* and positioned at *Bes* of ca. 13.8 eV, 17.0 eV, and 19.8 eV, correspondingly, are distinguished in both rGO and rGO–Th samples, being related to $K_{1}^{+}\left(\sigma_{3}\right)$ for feature *D*, converged $K_{3}^{+}\left(\sigma_{3}\right)$ and $\;M_{1u}^{+}\left(\sigma_{2}\right)$ states for feature *E*, and $M_{1g}^{+}\left(\sigma_{1}\right)$ for feature *F* [86,87]. Finally, the observed *Z* peak at *BE* of 24.8 eV and *C* peak at *BE* of 10.1 eV rise in the acquired spectra. The former one is related to the C 1*s* line excited by photons with energy of 390 eV due to incompletely damped diffraction third order, while the latter one originates from the electronic states of the σ(C=O) bonds in ketones and carboxyl groups [84]. It is worth noting that *C*′ peak centered at 11.1 eV in the GO VB spectrum is due to the mix of the aforementioned states of the σ(C=O) bonds in ketones and carboxyl groups with the states of the π(O-C-O) and σ(O-H) in carboxyls, which are abundant in highly oxidized GO. Despite this similarity, certain alterations are also to be distinguished. Particularly, a prominent *A*′ feature centered at *BE* of ~3.2 eV attributed to the contribution of the π states ($Q_{2u}^{-}$) of the graphene network is visible in the rGO VB spectrum [88], while being absent in the rGO–Th one. Hence, despite the high degree of the recuperation of the graphene π-network in rGO–Th, it is still lower than in rGO films prepared by high-temperature annealing, probably due to the local disruption of the π-conjugation by the introduced thiol groups. At the same time, no evidence of G′ peak located at *BE* of 27.5 eV and related to states originating from O 2*s* atomic orbitals of oxygen groups is noticed, which confirms the high degree of the GO reduction upon the applied thiolation method.

### 3.6. Chemiresistive Effect in rGO–Th

**Figure 6:**
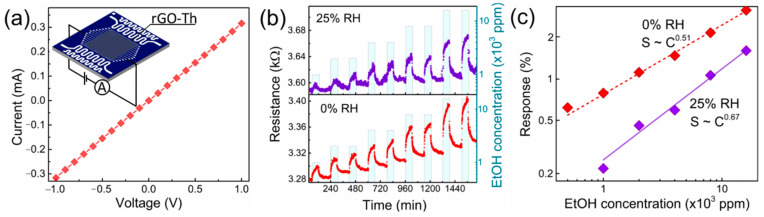
The chemiresistive performance of rGO–Th film. (**a**) I–V curve taken at both dc electric field directions under dry air conditions; the insert shows the measuring electrical circuit. Insert—a schematic model of the measured gas-sensor chip. (**b**) The resistance transient recorded under exposure to ethanol vapors of varied concentration C in range of 500–16,000 ppm when mixed with dry air (upper curve) and humid air (25% rel. humidity, bottom curve). (**c**) The dependence of chemiresistive response on the ethanol concentration in dry and humid air background. The measurements are taken at 40 °C to avoid environment temperature v ariations.

The observed functionalities make a promise for these rGO–Th films to be employed in gas sensors of chemiresistive type [89]. As detailed in the Section 2, the rGO–Th film was placed over a dielectric substrate with electrodes in the sealed chamber to measure the resistance at almost room temperature conditions, with *T* = 40 °C to be supported by meander heaters located at the substrate. Heating to such temperatures verified no effect of the surrounding conditions on the film chemiresistive properties. The deposition method provided a good Ohmic contact between the rGO–Th film and electrodes, as shown by the linear character of the I–V curve displayed in Figure 6a. It ensured the absence of significant potential barriers at the contact, which could mask the resistance of the film itself. The observed resistance values lie in the low kOhm range.

While exposing the film to ethanol vapors, the resistance steadily increases when compared to clean air conditions similar to the chemiresistive effect frequently observed in graphene or graphene oxide layers [12,31,89]. The Figure 6b shows the typical *R* (*t*) transient recorded upon ethanol exposures mixed with dry (0% RH) or humid (25% RH) air, for 60 min, interrupted by an intermediate purging of air. The ethanol concentrations were varied, at a 500–16,000 ppm range, repeating the film’s exposures to vapors of each concentration twice. As one can see, in both dry and humid air, the resistance follows the ethanol concentration with some residual drift of background. The chemisresistive response towards ethanol in the humid air is almost two times lower in comparison to the case of the dry air. We assert this to be related to the concurrent adsorption of the ethanol and water molecules on the rGO–Th, which limits the interaction between the ethanol and thiolated graphene and reduces the chemiresistive response.

Notably, both in dry and humid air, the resistance drops to almost initial values after the exposure to concentrations as high as 16,000 ppm without any external effect, namely heating or UV irradiation. The chemiresistive response evaluated according to Equation (1) is plotted versus the ethanol concentration in Figure 6c. The experimental data follow Freundlich’s isotherm of $S\sim C^{n}$ type, where *n* is equal to 0.51 ± 0.02 for the dry air and 0.67 ± 0.04 for the humid air. To compare the data obtained in the dry air with the literature ones, the sensitivity coefficient calculated as S/C was considered, yielding values in the range from 1.24 × 10^−3^%/ppm at low gas concentrations to 0.20 × 10^−3^%/ppm at concentrations of several tens thousands of ppm. The estimated sensitivity coefficient is lower than the ones observed earlier in thiol-functionalized rGO films, but taken in a N_2_ background that enhances the sensing performance [31]. Given the demonstrated functionality of the thiolated graphene in air ambient and its reproducible performance upon being exposed to exceptional concentrations of target gas, the rGO–Th films could be promising for further tests to develop room-temperature gas sensors for leak detection under real air conditions.

## 4. Conclusions

Summarizing, the facile method for the scalable production of thiolated graphene is developed with a report that reveals new details and peculiarities of the effect of the thiol groups on the morphology, electronic structure, and properties of the material. The synthesized thiolated graphene combines a functionalization by up to 5.1 at.%, which is among the highest reported concentrations, negligible amounts of other functionalities, and a high degree of restoration of the π-conjugated graphene network with the corresponding C/O ratio of 16.8. Accounting for such a chemistry and corrugated structure, originating from the corrugation of graphene layer upon thiolation and granting a high porosity, the synthesized thiolated graphene is regarded as a material of choice for electrochemical and catalytic applications, particularly when forming advanced platinum-on-carbon electrodes for fuel cells or metal-free porous electrodes for the oxygen reduction and oxygen evolution reactions.

A thorough analysis of the electronic structure, electrophysical, and optical properties of the thiolated graphene revealed that thiol groups have a negligible effect on the physics of the graphene layer, even at sufficiently high concentrations. This fact is reflected in an almost complete coincidence of optical absorption and conductivity characteristics as well as the work function value of the thiolated graphene when compared to the ones in a pristine rGO. Furthermore, an accurate examination of the VB of the graphene revealed the lack of appearance of localized electronic states upon the thiolation. Combined with the high reactivity of thiols, the pointed out VRH mechanism of conductivity with the finite sheet resistance values of 2–3 KΩ/sq along with the optical transparency makes thiolated graphene an attractive material for use as a sensing layer in chemiresistive and optical bio- and gas sensors. The application of rGO–Th as a promising gas-sensing material is supported by the performed preliminary tests.

Taken together, the developed method and findings of the morphology and physical properties of thiolated graphene guide and outline the frames of this graphene derivative’s application in advanced sensing, optoelectronic, and energy conversion technologies as well as giving a hint on the features of the alteration of graphene physics upon derivatization.

## Figures and Tables

**Table 1:** Composition of functional groups (at.%) and C/O ratio in GO, rGO–Br, and rGO–Th derived from the processed C 1*s* spectra.

Component	C–V	C=C	C–C	C-OH & C-O-C	C=O	COOH/-SO_x_^2−^	C–Br/C–S	C/O Ratio
Binding Energy (eV)	283.9	284.7	285.1	286.8	288.1	289.0	286.1	
GO	1.16	50.62	5.40	37.30	4.26	1.26	-	2.3
rGO–Br	2.42	89.43	<0.01	<0.01	2.68	0.04	5.43	37.8
rGO–Th	<0.01	89.39	1.67	<0.01	1.46	2.25	5.23	16.8

## Data Availability

The data presented in this study are available on request from the first author.

## Supplementary Materials

The following supporting information can be downloaded at: https://www.mdpi.com/article/10.3390/nano12010045/s1. Section S1: The description of the setup for the gas sensing measurements; Figure S1: The scheme of the experimental setup to study the chemiresistive response of rGO-Th gas sensor; Figure S2: Size distribution histograms acquired from the laser diffraction measurements of the (a) GO and (b) rGO-Th suspensions 0.05 wt.% of concentration; Figure S3: Scanning electron microscopy (SEM) low-magnification images of the (a) rGO-Br and (b) rGO-Th layers; Figure S4: SEM high-magnification images of the (a) rGO-Br and (b) rGO-Th platelets; Figure S5: (a) Transmission electron microscopy (TEM) image and (b) Electron diffraction pattern rGO-Br individual platelet.
