# Supplementary material for: A Blueprint for the Synthesis and Characterization of Thiolated Graphene
Source: Nanomaterials (Basel). 2021 Dec 24;12(1):45. doi: 10.3390/nano12010045 (PMC8746421; doi:10.3390/nano12010045)
Supplement: Supplementary file 1 [file nanomaterials-12-00045-s001.zip › nanomaterials-1497038-supplementary.pdf]

Supplementary Materials

# A Blueprint for the Synthesis and Characterization of Thiolated Graphene

**Maxim K. Rabchinskii [1,*], Victor V. Sysoev [2], Sergei A. Ryzhkov [1], Ilya A. Eliseyev [1], Dina Yu. Stolyarova [3], Grigorii A. Antonov [1], Nikolai S. Struchkov [4], Maria Brzhezinskaya [5], Demid A. Kirilenko [1], Sergei I. Pavlov [1], Mihail E. Palenov [6], Maxim V. Mishin [6], Olga E. Kvashenkina [6], Pavel G. Gabdullin [6], Alexey S. Varezhnikov [2], Maksim A. Solomatin [2] and Pavel N. Brunkov [1]**

1 Ioffe Institute, Politekhnicheskaya St. 26, 194021 Saint Petersburg, Russia; ryzhkov@mail.ioffe.ru (S.A.R.); Ilya.Eliseyev@mail.ioffe.ru (I.A.E.); antonov@mail.ioffe.ru (G.A.A.); Demid.Kirilenko@mail.ioffe.ru (D.A.K.); Pavlov_sergey@mail.ioffe.ru (S.I.P.), Brunkov@mail.ioffe.ru (P.N.B.)
2 Department of Physics, Yuri Gagarin State Technical University of Saratov, 77 Polytechnicheskaya St., 410054 Saratov, Russia; vsysoev@sstu.ru (V.V.S.); alexspb88@mail.ru (A.S.V.); solomatin1994@gmail.com (M.A.S.)
3 National Research Centre "Kurchatov Institute", Akademika Kurchatova pl. 1, 123182 Moscow, Russia; stolyarova.d@gmail.com
4 Center for Probe Microscopy and Nanotechnology, National Research University of Electronic Technology, Bld. 1, Shokin Square, 124498 Moscow, Russia; struchkov.nikolaj@gmail.com
5 Helmholtz-Zentrum Berlin für Materialien und Energie, Hahn-Meitner-Platz 1, 14109 Berlin, Germany; maria.brzhezinskaya@helmholtz-berlin.de
6 Institute of Electronics and Telecommunications, Peter the Great St.Petersburg Polytechnic University (SPbPU), Polytechnicheskaya 29, 195251 Saint Petersburg, Russia; m.e.palenov@gmail.com (M.E.P.); max@mail.spbstu.ru (M.V.M.); kvol.spbspu@gmail.com (O.E.K.); gabdullin_pg@spbstu.ru (P.G.G.)
* Correspondence: rabchinskii@mail.ioffe.ru

## Section S1. The Setup for the Gas Sensing Measurements

The multielectrode chip with rGO-Th film was placed into the 50-pin ceramic holder. Co-planar electrodes on the chip were connected to corresponding Au contact pads on the holder via ultrasonic welding (WEST Bond, USA) using Au microwires, 38 μm of thickness.

The chemiresistive response of the rGO-Th film was analyzed in relation to vapors of ethanol. The gas-sensing parameters were studied in the mixture with both humid (humidity level is ~25 rel.%) and dry (humidity level is ~0 rel.%) air. The background humid-zero air was provided by dry air generator (PG14L, Peak Scientific, UK) employing a filtered lab air (Figure S1). The humidity level in the delivered gas was adjusted by passing the dried air via a distilled water-filled bubbler; the control of humidity level at the background was maintained by mixing the dry air and $H_2O$ 100 rel.%-rich air at the installed ratio employing high-precision flow meter controllers (Bronkhorst, The Netherlands). The independent verification of humidity level in the gas flow was provided with a commercial humidity sensor of capacitance type (AM2302, Aosong, China) interfaced with PC. The analyte organic vapors were delivered to the gas pipelines from a bubbler containing the corresponding solution which was forked with a line containing the dry air. The input pressure to the gas-mixing setup was provided with

a compressor (Peak Scientific, UK) with pipelines equipped with two-way and three-way remote valves. All the gas-mixing setup was driven by PC.

The concentration of the target analytes in the carrier gas at the outlet from the bubbler was calculated using the following equation:

$$\mathbf{C} = \frac{\mathbf{P_{sat}}}{\mathbf{P_{atm}}}, \quad (1.1)$$

where $P_{sat}$ is a saturation vapor pressure and $P_{atm}$ is the atmospheric pressure. The value of atmospheric pressure is used because the outlet part of our gas mixing system is not blocked by any valve and the outlet gas ere free exhausted.

To obtain lower concentrations than the one of saturated vapors, a gas mixture dilution scheme was used using an additional flow controller. To calculate the concentration value in this case, the following equation was applied:

$$\boldsymbol{C} = \frac{\boldsymbol{F_{gas}} \times \boldsymbol{P_{sat}}}{\boldsymbol{F_{gas}} \times \boldsymbol{P_{sat}} + (\boldsymbol{P_{atm}} - \boldsymbol{P_{sat}}) \times \boldsymbol{F_{gas}} + \boldsymbol{P_{atm}} \times \boldsymbol{F_{air}}} \quad (1.2)$$

where $F_{gas}$ is the flow rate through the bubbler ($cm^3$/min), $F_{air}$ is the flow rate of the diluent gas ($cm^3$/min), air in our case, $P_{sat}$ is the saturated vapor pressure of the bubbling solution of analyte (in mm Hg), and $P_{atm}$ is the atmospheric pressure (in mm Hg). The resulting value obtained from the (1.2) equation is the ratio of the molecules of analyte from the bubbling solution to the air particles. By multiplying this value by 1000000 (i.e. per million air particles), we can get the result in ppm. Note that the (1.2) equation is universal, and in the case of $F_{air} = 0$, corresponding to the absence of a flow of diluent gas, it turns into (1.1) equation which refers to the case of saturated vapors of analyte going from the bubbler.

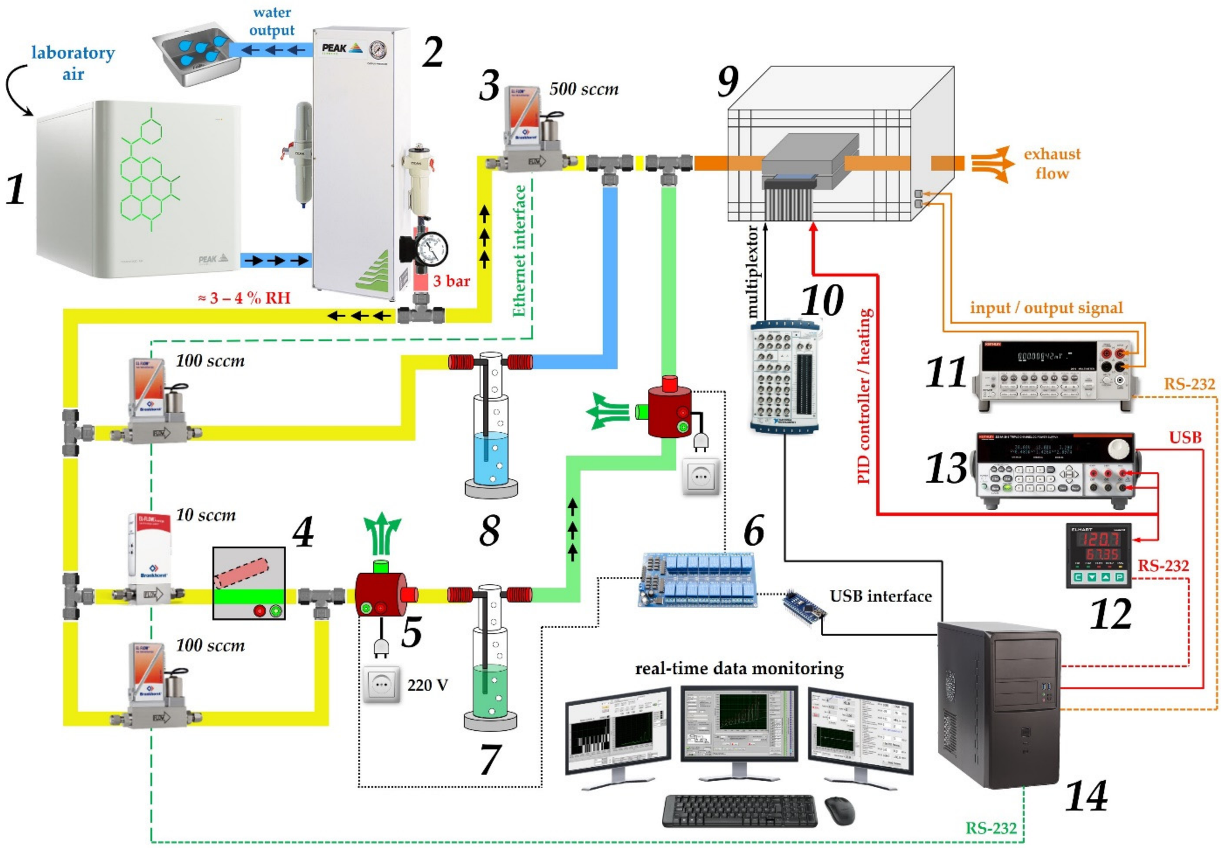


**Figure S1.** The scheme of the experimental setup to study the chemiresistive response of rGO-Th gas sensor. 1 – compressor; 2 – filter dryer; 3 – precise mass-flow controller; 4 – two-way valve; 5 - three-way valve; 6 – relay controlling the valves; 7 – a bubbler containing the analyte; 8 - a bubbler containing the water; 9 – Faraday cage containing the chip mounted into a sealed stainless-steel chamber; 10 - data acquisition platform NI-DAQ (National Instruments, USA); 11 – Multimeter Keithley-2000, (Keithley Instruments, USA); 12 – Elhart PID controller; 13 – Heating power supply Keithley-2231A-30-3 (Keithley Instruments, USA); 14 – PC managing the measurements.

**Section S2. Size Distribution Measurements Employing Laser Diffraction Method**

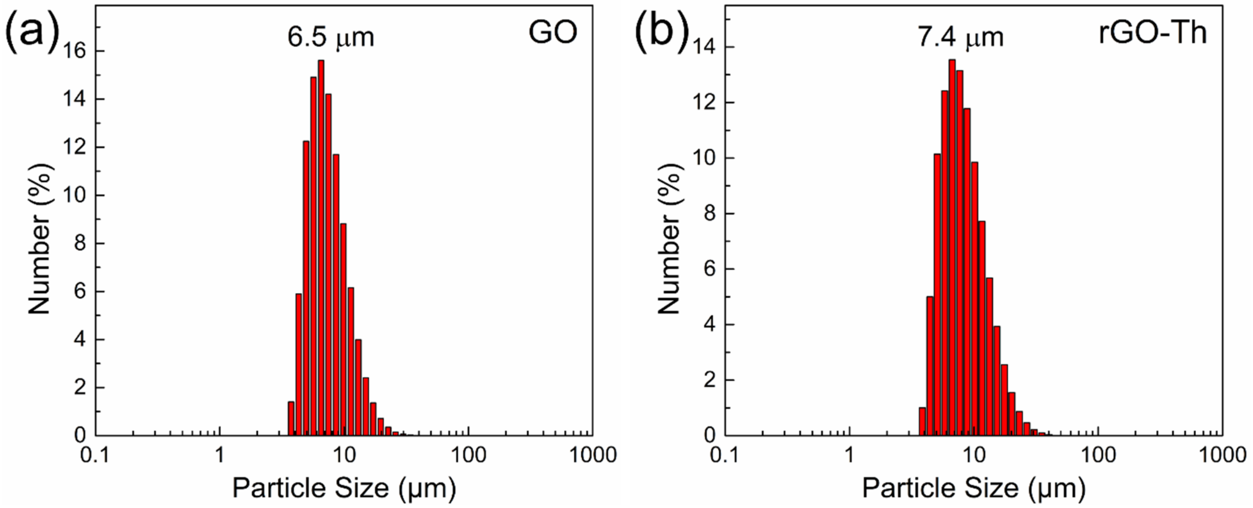


**Figure S2.** Size distribution histograms acquired from the laser diffraction measurements of the (**a**) GO and (**b**) rGO-Th suspensions 0.05 wt.% of concentration

**Section S3. Morphology of rGO-Br**

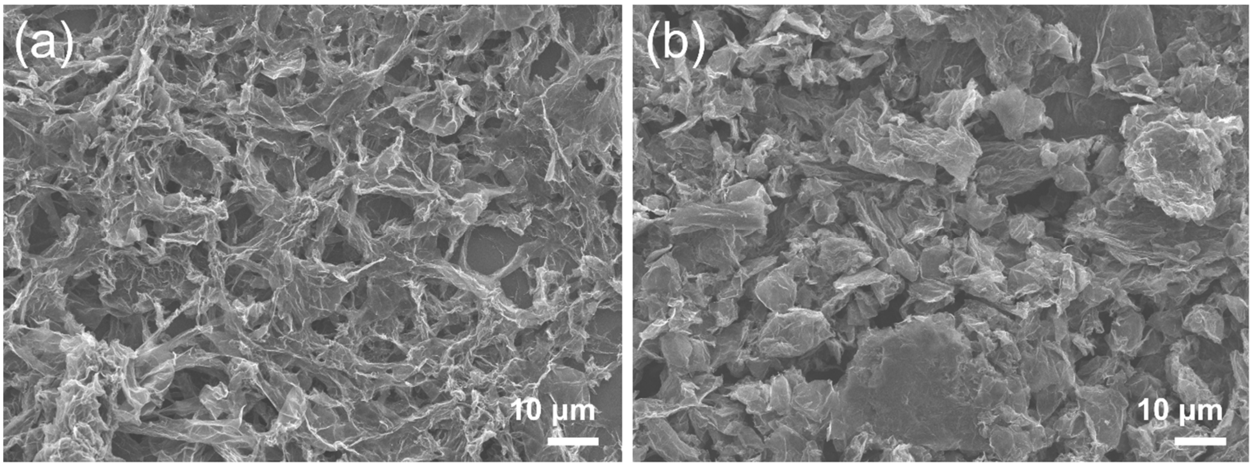


**Figure S3.** Scanning electron microscopy (SEM) low-magnification images of the (**a**) rGO-Br and (**b**) rGO-Th layers.

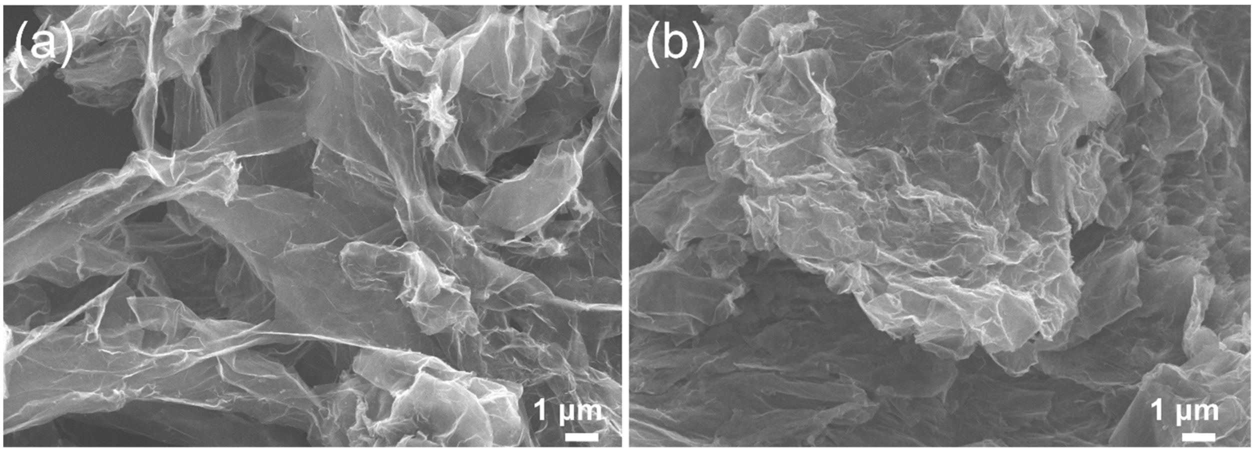


**Figure S4.** SEM high-magnification images of the (**a**) rGO-Br and (**b**) rGO-Th platelets.

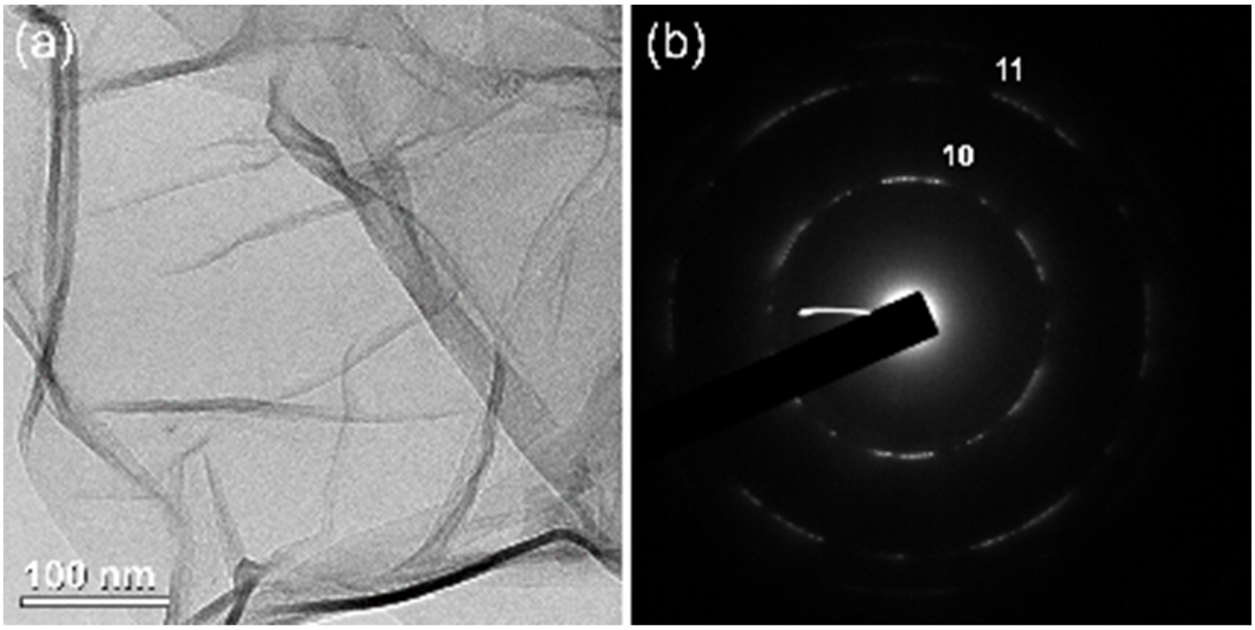


**Figure S5.** (**a**) Transmission electron microscopy (TEM) image and (**b**) Electron diffraction pattern rGO-Br individual platelet.
